# Investigating interpretation bias and stress responses as risk factors in children of parents with depression

**DOI:** 10.1186/s12888-026-08408-z

**Published:** 2026-07-29

**Authors:** Tonya Frommelt, Leonie Bäumler, Nicolas Rohleder, Anna Bartosch, Lily Schneider, Charlotte Sumpf, Anca Sfärlea, Gerd Schulte-Körne, Belinda Platt

**Affiliations:** 1 Department of Child and Adolescent Psychiatry, Psychosomatics and Psychotherapy, LMU University Hospital Munich, Nußbaumstr. 5, 80336 Munich, Germany; 2 Department of Psychiatry and Psychotherapy, LMU University Hospital, Munich, Germany; 3 Chair of Health Psychology, Department of Psychology, Friedrich- Alexander- Universität Erlangen-Nürnberg, Erlangen, Germany

**Keywords:** Parental depression, High-risk youth, Interpretation bias, Salivary cortisol, Cortisol reactivity, Cortisol recovery, Trier Social Stress Test for Children (TSST-C), Affective reactivity, Depression, Anxiety

## Abstract

**Background:**

Children of parents with depression, hereafter referred to as high-risk youth, are at elevated risk for mental illness, yet the mechanisms underlying this vulnerability remain unclear. Biased interpretation of ambiguous information and altered stress responses in this population have been proposed as potential pathways. As no previous study has examined these factors together, we investigated interpretation bias and endocrinological and affective stress responses as potential markers of depression vulnerability in high-risk children. We hypothesised that children of parents with depression would show more negative interpretation bias, altered cortisol stress responses and greater affective reactivity and prolonged recovery compared to children of parents with no mental illness. Across the sample, associations between interpretation bias and stress responses were expected.

**Method:**

Participants were 80 high-risk (parental depression) and 77 low-risk (no parental mental illness) youth aged 10 to 14 years. Mental health was assessed using structured clinical interviews. Interpretation bias was measured using the Scrambled Sentences Task, and stress reactivity and recovery were indexed by salivary cortisol and self-reported affect across six time points. Group differences were tested with *t*-tests and analyses of covariance. Regressions examined the unique contribution of familial risk status beyond other relevant factors, including depression, anxiety, childhood trauma, pubertal status, and sex. Correlations examined associations between interpretation bias and cortisol and affective stress indices.

**Results:**

Groups did not differ in interpretation bias, or cortisol or affective stress responses. Children’s own symptoms of psychopathology (depression slightly more so than anxiety) positively predicted their interpretation bias but not their cortisol or affective stress responses. Exploratory moderation analyses showed that higher baseline cortisol strengthened the relationship between depression and interpretation bias. Interpretation bias correlated with cortisol reactivity and recovery within the high-risk but not the low-risk group.

**Conclusion:**

Contrary to theoretical accounts, high-risk youth did not exhibit cognitive, affective, or endocrinological vulnerabilities relative to low-risk peers. Interpretation bias was found to be a promising target, robustly linked to clinical outcomes and tentatively to cortisol stress responses, though its absence in high-risk youth is in support of a more selective intervention approach.

**Trial registration:**

The study was preregistered under the Deutsches Register Klinischer Studien DRKS00028842 on August 19, 2022.

**Supplementary Information:**

The online version contains supplementary material available at 10.1186/s12888-026-08408-z.

## Introduction

Depressive disorders are among the most prevalent mental health conditions affecting children and adolescents worldwide. An international meta-analysis found that approximately 21.3% of children and adolescents experience at least mild symptoms of depression, with around 3.7% meeting criteria for major depressive disorder (MDD) [1]. Children of parents with depression (hereafter referred to as high-risk; HR) are at substantially elevated risk, being 2.3 times more likely to develop depression and 1.9 times more likely to develop any mental disorder, compared to low-risk (LR) peers without such a familial history [2]. Child and adolescent MDD has been found to lead to greater episode frequency and duration, increased hospitalisation and suicidality rates and higher psychiatric comorbidity relative to adult-onset MDD [3–5]. The World Health Organisation (WHO) emphasises the need for improved preventive interventions [6]. However, existing interventions targeting HR children show limited effect sizes, underscoring the need for a better understanding of the transgenerational transmission of depression [7].

Children of parents with mood disorders without a history of mental illness themselves represent a critical group for investigating early risk pathways. Theoretical frameworks propose that elevated risk in HR youth emerges from the interplay of biological, cognitive, affective, and behavioural vulnerabilities for depression [8]. Although it is thought that vulnerabilities are likely to interact and influence one another [8, 9], few studies have examined the relationships between these different levels of vulnerability in HR youth. The current study addresses two of the most established vulnerability factors for depression amongst HR youth: negative cognitive biases and cortisol and affective responses to stress. Examining their association is important, because the presence of cognitive biases alone does not necessarily justify their selection as a target for preventive intervention. Rather, such biases only represent meaningful intervention targets if they contribute to downstream risk processes, for example by shaping stress responses or otherwise increasing vulnerability to later psychopathology.

Negative cognitive biases can be defined as the tendency to preferentially attend to and process negative rather than neutral or positive information. While such biases can manifest in the form of attention bias (AB), interpretation bias (IB) and memory bias (MB) [10, 11], this study explores the effects of IB [12], given its more robust association with depression [13, 14] relative to AB [15] and MB [16], especially in youth [17]. Negative IB has been consistently observed in both adults [12, 18] and youth [17] with depression. Importantly, IB seems to be related to symptoms of depression not only cross-sectionally but also longitudinally, as a recent meta-analysis of 81 studies found IB to prospectively predict symptoms of depression in both youth and adults [14]. In accordance with the diathesis-stress model, this appears to be particularly true for individuals who experience stressful life events, consistent with findings from previous studies in both children [13] and adults [19, 20]. Assessing such biases is therefore most appropriate following stress induction, as these vulnerabilities may otherwise remain less readily observable. Negative IB has also been found in adults with subclinical symptoms when compared to never-depressed adults, suggesting it may play a role in the risk for developing future depressive episodes [21]. Studies with HR children found them to interpret emotionally ambiguous words, stories [22] and sentences [23] more negatively than their LR peers. According to the perseverative cognition hypothesis, such persistent negative thinking can prolong activation of the hypothalamic-pituitary adrenal (HPA) axis, thereby sustaining physiological stress responses and contributing to the development of mood disorders [24, 25].

The HPA axis is a central neuroendocrine system underlying the body’s response to stress. Its end product, cortisol, supports adaptive regulation under stress, although prolonged activation may contribute to allostatic load and affect emotion-related brain regions [26, 27]. HPA activity can be characterised by basal levels, immediate reactivity to stress, and post-stress recovery [26]. Empirical evidence on endocrinological stress reactivity and recovery in HR samples has yielded complex findings. A recent systematic review found heightened basal cortisol as well as a tendency for elevated cortisol reactivity to stress in HR compared to LR children [28], with a subset of studies also investigating and finding sustained cortisol in the recovery period (29–32]. Similarly, previous research reported that HR adults showed greater cortisol reactivity to a stress test compared to controls, and these group differences could not be explained by children’s symptoms of depression, anxiety, or negative life events [33]. In contrast, further research found HR adolescents to show elevated dehydroepiandrosterone/cortisol ratios, suggesting HPA axis blunting, but only at high levels of reported childhood trauma [34]. Blunted cortisol responses have also been linked to self-reported childhood adversity in adults [35]. Another study found blunted reactivity in adolescent HR females and typical trajectories in adolescent HR males [36]. Moreover, the onset of MDD has been predicted by cortisol hypo-reactivity in girls at earlier stages of pubertal development and by cortisol hyper-reactivity in girls at more advanced pubertal stages [37]. Similarly, previous research found a switch from cortisol hypo- to hyper-reactivity among at-risk, dysphoric youth as a function of pubertal development [38]. A further HR-LR youth study reported null effects [39]. In summary, findings on the relationship between depression risk and cortisol reactivity and recovery may be influenced by factors such as sex [37, 39–44], pubertal development [37, 41] and childhood adversity [34, 45, 46]. These findings underscore the complexity of HPA axis functioning. While elevated endocrinological stress reactivity has been proposed as a biomarker for depression risk [36], the relationship may not be as straightforward as suggested, highlighting the need for greater clarity on how stress presents in HR youth populations and its relationship with other vulnerability factors.

A longitudinal study showed that youth from low socioeconomic backgrounds exhibited higher average levels of IB over time, and these appraisals mediated the association between socioeconomic status and cardiovascular reactivity [47]. Another study reported that IB, but not AB, moderated the relationship between group status (clinically anxious versus typically developing youth) and sympathetic arousal, as measured by skin conductance during the Trier Social Stress Test for Children (TSST-C) [38]. A final study, found IB to be related to blunted cortisol stress reactivity in adolescents with Autism Spectrum Disorder [48]. Very few studies have investigated the association between cognitive and physiological vulnerability in a HR sample. Moreover, none of the available HR studies have investigated the association between stress responses and IB specifically, although recent findings suggest that IB is more reliably associated with HR populations than other cognitive biases [13]. One HR study found that parental depression and child cognitive vulnerability jointly predicted children’s cortisol reactivity, with elevated cortisol levels observed in those exhibiting depressogenic attributions and AB or MB [49]. A second study demonstrated that modifying AB in a HR adolescent sample buffered against stress-induced increases in negative affect and physiological arousal [50]. One RCT, albeit in a LR adolescent sample, demonstrated that altering IB can influence affective reactivity, such that elevations in negative affect were found when training negative IB but no effect on affect was found when training positive IB [51]. A further RCT in LR adolescents found that positive IB training was associated with reductions in negative affect; however, the manipulation check did not confirm a significant change in IB post training, making the validity of these findings questionable [52]. Together, these studies provide preliminary evidence for a link between cognitive vulnerability and stress responses, although the findings require further replication and clarification particularly in a HR sample.

In this manuscript, we investigate the presence of IB and altered stress responses in HR youth and whether these two risk factors are associated with one another. The present study is the first to examine IB and stress reactivity and recovery simultaneously in HR youth. This study is the baseline assessment for a randomised controlled trial (RCT), which in future phases will explore IB as a mechanism of maladaptive endocrinological and affective stress reactivity via a four-week cognitive training program [53].

**Fig. 1:**
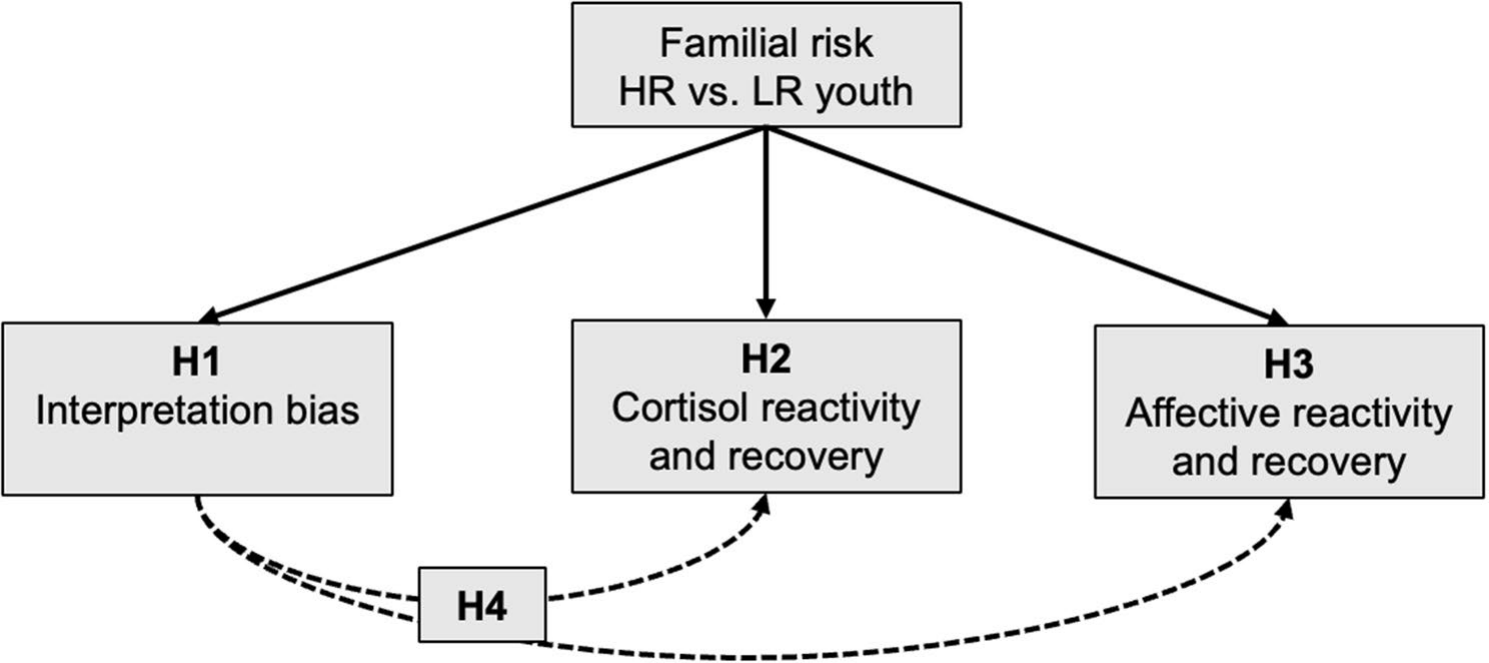
Schematic overview of hypotheses. *Note.* Solid lines represent hypothesised group differences; dotted lines represent hypothesised associations between variables across groups; HR = high-risk participants (children of parents with depression); LR = low risk participants (children of parents with no history of mental illness)

We hypothesise that HR children will exhibit (i) more negative IB, (ii) altered cortisol reactivity and recovery, and (iii) greater affective reactivity and worse affective recovery in response to stress compared to LR peers. Moreover, we hypothesise that (iv) across both groups, IB and stress responses will be associated. In the present cross-sectional study, we examine whether these processes are interrelated rather than testing their temporal sequence. Although theoretical models such as the perseverative cognition hypothesis suggest that IB may contribute to altered stress responses, the temporal ordering of these processes should be addressed in future longitudinal research. Moreover, given inconsistencies in previous findings regarding the direction of cortisol reactivity and recovery, we refrain from making directional predictions about endocrinological findings, that is, whether HR participants will show heightened or attenuated cortisol responses. This exploratory approach allows us to examine whether cognitive and biological markers are jointly altered in HR youth and may reflect an integrated vulnerability profile for depression risk. Figure 1 depicts a schematic overview of the hypotheses tested.

## Methods

### Participants and design

This study received ethical approval from the Ludwig-Maximilians-University (LMU) Medical Division Ethics Committee, Munich, Germany (Study ID: 19–691). The final sample included in the current study consisted of 157 participants, of which *n* = 77 were considered LR and *n* = 80 were considered HR. Participants were between 10 and 14 years of age. This age range was selected as, below age 10, children may have greater difficulty completing the cognitive tasks, whereas during mid-adolescence the incidence of depressive episodes begins to rise sharply [54], such that restricting the sample to younger participants increased the likelihood of capturing risk processes prior to disorder onset. Participants had at least one parent who fulfilled the diagnostic criteria for a current or past major depressive episode (HR) or no parent with a history of depression or other mental illnesses (LR). Participants were excluded if they themselves met the diagnostic criteria for a current or past episode of mental illness. High-risk participants were excluded if either parent had a history or current diagnosis of bipolar disorder, psychosis, or substance abuse. We decided to include participants with a history of enuresis, provided that their symptoms were not indicative of any underlying psychological conditions. Other comorbid disorders in the parents were allowed, provided that the depressive diagnosis was deemed primary: approximately 14% of parents in the HR group met the criteria for a past or present comorbid mental illness including anxiety disorders (*n* = 16), PTSD (*n* = 3) and other (*n* = 3). Additionally, participants were required to have proficient German-language skills due to the verbal nature of various components of the study.

Standardised, semi-structured clinical interviews were conducted to assess mental health diagnoses in parents (Diagnostisches Interview bei psychischen Störungen; DIPS) [55] and in children (Diagnostisches Interview bei psychischen Störungen im Kindes-und Jugendalter; K-DIPS) [56]. The DIPS and K-DIPS are widely used German diagnostic interviews that allow for the assessment of a broad range of Axis I psychiatric disorders according to DSM-5 criteria. Both instruments have shown strong interrater reliability, with agreement rates of at least 87% across all diagnoses [57, 58]. The interviews were conducted and analysed by a person who was trained in conducting the DIPS / K-DIPS. Out of 218 families screened, 61 were excluded post screening and pre-testing due to a parent or children fulfilling the exclusion criteria. Twenty-four exclusions were attributable to children meeting criteria for a diagnosis, with anxiety disorders (*n* = 14), ADHD/ADD (*n* = 7), and depressive disorders (*n* = 4) representing the primary categories (note: numbers exceed 24 due to comorbidities). Three additional children were taking medication with potential relevance for cortisol levels. Of the two children taking the birth control pill, one showed no altered cortisol levels, while the other showed elevated overall cortisol but a typical response pattern, with cortisol increasing and decreasing as expected; statistically exclusion was not considered necessary. The third child, who was taking thyroid and sleep medication, was excluded from the study. Interrater reliability was determined for 20% of the sample (*n* = 33 parents and *n* = 33 children) by an independent researcher who re-rated the diagnostic interviews. Agreement on inclusion/exclusion decisions based off the specific criteria relevant to the individual was 93.93% for both parents and children.

High-risk and LR children differed in age, with LR participants being 5.2 months older on average. Participants were financially compensated for their time and had their travel expenses reimbursed. Participants received €25 for attending the screening session, which was conducted either via video call or in person at the university hospital research department according to participant preference, and an additional €25 for completing the testing session, which was always conducted in person. To address recruitment challenges and make use of available discretionary funds, the compensation for the testing session was later increased to €40. Both parents (if applicable) and child provided written informed consent. All testing sessions took place in the research department of the Child and Adolescent Psychiatry of the LMU University Hospital in Munich.

### Recruitment

Although several recruitment avenues were explored, most participants (approximately 84%) were recruited through personalised letters and study flyers distributed via district administration offices to families with children in the target age range. This ensured a largely homogeneous recruitment process. Additional participants were recruited through referrals from therapists in Munich and surrounding areas, re-contacting participants from previous departmental studies who had consented to future participation, distributing flyers to local schools, and placing posters and flyers in public locations such as clinics and private practices. Recruitment efforts also extended to social media, including Instagram advertising, posts in relevant Facebook groups, and content shared on the research group’s own channels. All participants came from the greater Munich area.

### Procedure

**Fig. 2:**
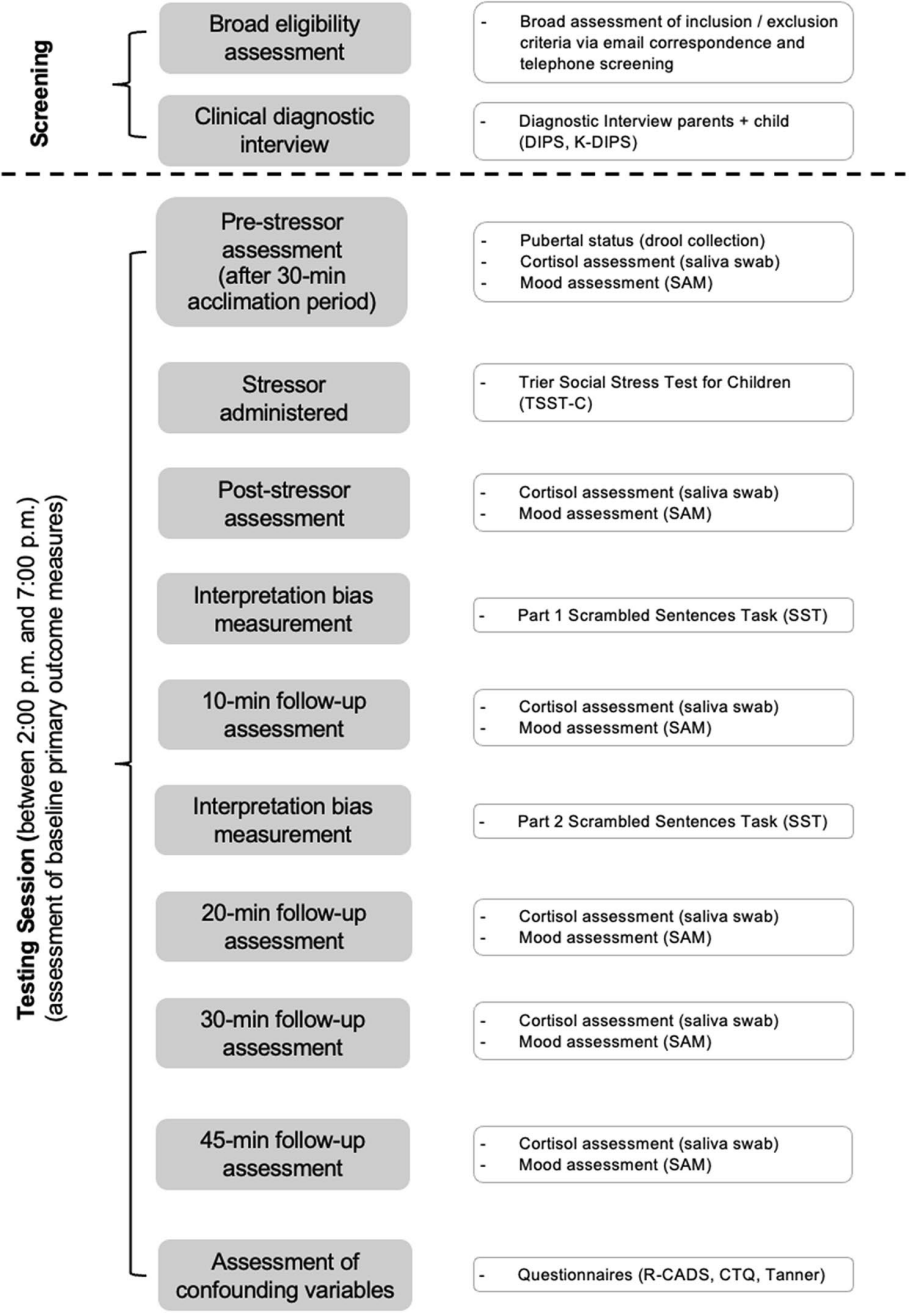
Schematic overview of the study procedure. *Note*. CTQ = Childhood Trauma Questionnaire; DIPS = Diagnostic Interview for Mental Disorders; K-DIPS = Diagnostic Interview for Mental Disorders in Children and Adolescents; R-CADS = Children’s Anxiety and Depression Scale; SST = Scrambled Sentences Task; SAM = Self-Assessment Mannikin Scale

See Fig. 2 for an overview of the study procedure. Families were initially broadly screened for basic inclusion/exclusion criteria by email and telephone. Eligible families then underwent the clinical diagnostic interview. Parents and children were also questioned on participants’ current medication use, as this can interfere with cortisol levels. Initially, the clinical diagnostic interview was conducted with children participating in the study and both parents. However, after receiving feedback from families about the extensive time commitment required, we modified our screening process to conduct the clinical interview with only one parent. The second parent’s mental health was then broadly evaluated through an external assessment based on information provided by the participating parent. If one parent was affected by mental illness (in the HR group) or was suspected to have mental health issues (in the LR group), the clinical interview was always conducted with this parent. In the days following the screening session the researchers involved in the screening procedure discussed the eligibility of the family and a final decision was communicated to them.

The testing session was scheduled no more than 12 weeks after screening where data on participants’ IB, affective and endocrinological stress reactivity and recovery as well as confounding variables was collected (see Table S7 in Sect.  3 of the Supplemental Materials for an overview of all measures). Participants were instructed to refrain from eating for one hour prior to testing and to refrain from drinking 10 min prior to testing to minimise cortisol interferences. Sessions were scheduled between 2:00 and 7:00 pm to ensure feasibility through a practical time window while minimising time-of-day effects [59]. Upon arrival, participants had a 30-minute acclimatisation period, during which saliva samples were collected for hormone assessment and participants engaged in a non-stressful activity of choice such as reading. At the 30-minute time point, baseline affect and cortisol levels were assessed. Next, the TSST-C [60] was administered. Immediately after the TSST-C, stress responses were assessed for the second time. During the 10-minute break between the second and third stress assessments, part 1 of the Scrambled Sentences Task (SST)[61] was completed. At 10 min post TSST-C, stress responses were assessed for the third time. Between stress assessments three and four, part 2 and 3 of the SST were completed. Stress responses were assessed for the fourth, fifth and sixth time at 20-, 30- and 45-minutes post TSST-C. The SST was administered after the TSST-C to assess IB in a stress-activated context, consistent with cognitive theories proposing that such vulnerabilities may remain latent until activated by stress. Because stress response assessments occurred at fixed time points and the SST could not be completed within a single interval between them, the task was interleaved with these assessments and therefore administered in two parts. Finally, data on potentially confounding variables, namely childhood trauma, depression and anxiety symptoms as well as sexual maturation ratings were collected. After study completion, participants were fully debriefed about the TSST-C. They were informed that their performance would not be compared to others in the study, that the audience was staged, and that the task was intentionally designed to induce stress. They also received positive feedback on their performance.

### Outcome measures

#### Interpretation bias

Interpretation bias was assessed using a computerised version of the SST which was constructed using *Inquisit* (2022) [62]. Two randomised versions (A and B) of the SST were administered. Participants were presented with 36 scrambled sentences; each containing six words and were instructed to construct meaningful sentences using five of these words by numbering the words in a meaningful order as quickly and accurately as possible. Sentences were presented for a duration of 18 s before the next sentence automatically appeared. Of the total, 30 sentences were emotionally ambiguous (e.g., “total I winner a loser am”) with both positive and negative solutions, while six were neutral (e.g., “like watching funny I exciting movies”) with neutral solutions only. Version A of the SST was based on sentences from an earlier study by members of the current research team [23]. These sentences originated from the original stimulus set [61], were translated into German [63] and then adapted and extended for the study [23]. Version B, created specifically for this study, largely mirrored version A, with 93% of the emotional sentences modified slightly by altering individual words (e.g. to change the setting of the scene or by selecting novel emotionally valanced words). The remaining 7% of emotional sentences in version B were directly adopted from another study [64]. To prevent participants from intentionally strategizing during sentence formation, a simultaneous cognitive load task was administered [10]. Participants were initially presented with a fixation cross and then a four-digit number for 5,000ms at the start of each of three blocks. Participants should memorise and then recall the number at the end of each block. Responses from the SST were categorised as grammatically correct negative, grammatically correct positive or other. Interpretation bias scores were computed as the proportion of negative interpretations out of all grammatically correct emotional sentences. Sentences were manually rated by two independent raters, and the average proportion was calculated as the final score.

**Fig. 3:**
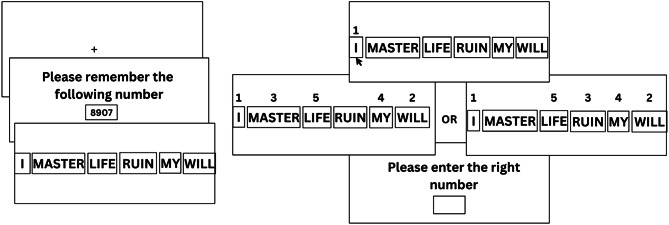
Schematic overview of the scrambled sentences task trial structure. *Note*. Top left: fixation cross; Middle left: presentation of the number to be recalled at the end of the block (cognitive load task); Bottom left: scrambled sentence display; Top right: first mouse click indicating the selected starting word; Middle right: positive and negative sentence resolution options; Bottom right: recall of the number from the cognitive load task

A meta-analysis on 93 studies found the SST to have good reliability and good convergent validity [65]. To examine whether the two SST versions (A vs. B) differed in the present study, an independent-samples *t*-test was conducted. Results indicated no significant difference in mean scores between Version A (*n* = 76, *M* = 0.119, *SD* = 0.133) and Version B (*n* = 81, *M* = 0.120, *SD* = 0.121), *t*(155) = − 0.02, *p* = .986, 95% CI [–0.040, 0.040]. The effect size was negligible (Cohen’s *d* = − 0.003). See Supplemental Materials Sect.  1 (Tables S1.1, S1.2, S2.1 and S2.2) for an English translation of the sentences used. See Fig. 3 for a schematic overview of the SST trial structure.

#### Stress reactivity and recovery

To measure stress responses, the TSST-C, a validated protocol for eliciting stress in children aged 8 to14 years, was used. The procedure involved two five-minute tasks: verbal storytelling and performing serial subtraction in front of a panel of two observers. The TSST-C is an established method for eliciting stress responses and is adapted from the adult TSST, which reliably triggers a two- to fourfold cortisol increase [66]. A second TSST-C version was developed for the follow-up session of the RCT component of the study, which is not reported in the present paper, to match the original version while minimising repetition effects through novel story and subtraction tasks [53]. Allocation of Version A or B to the baseline versus follow-up session was random. Affective responses to stress were measured using the Self-Assessment Manikin (SAM) [67], a 9-point pictorial scale depicting affective valence from highly negative to highly positive. Affective ratings were collected simultaneously to saliva samples (see Fig. 2). The SAM has demonstrated strong correlations with more extensive verbal mood assessments, offering an efficient means of tracking affective reactivity [67]. Analyses of covariance (ANCOVAs) indicated no significant differences between Version A and Version B of the TSST-C in either cortisol or affective reactivity, operationalised as the highest cortisol level or lowest mood rating within 30 min following the stressor, controlling for baseline cortisol and affect, respectively. For cortisol, reactivity did not differ between Version A (*n* = 84, *M* = 1.49, *SD* = 0.86) and Version B (*n* = 72, *M* = 1.37, *SD* = 0.90), *F*(1, 153) = 0.001, *p* = .971, partial *η²* < 0.001. Similarly, for affective reactivity, Version A (*n* = 84, *M* = 5.94, *SD* = 1.59) did not differ significantly from Version B (*n* = 73, *M* = 5.73, *SD* = 1.19), *F*(1, 154) = 0.18, *p* = .676, partial *η²* = 0.001.

### Covariates

#### Pubertal status

Pubertal development was evaluated using the self-report version of the Tanner Sexual Maturation Rating (SMR) [68, 69]. Participants were shown gender-specific illustrations of secondary sexual characteristics and asked to select the image that best reflected their current developmental stage. They rated pubic hair development on a 5-point scale and, depending on sex, rated either breast (female) or genital (male) development on a 5-point scale. The two ratings were averaged to derive a SMR. To complement self-reports, salivary samples were collected from a subsample of participants (*n* = 111) to assess estradiol, progesterone, and testosterone levels. Tanner SMRs were validated against these hormone measures.

#### Symptoms of anxiety and depression

To control for potential confounding effects of internalising symptoms on IB and cortisol responses, children’s depression and anxiety symptoms were assessed using the German version of the Revised Children’s Anxiety and Depression Scale (R-CADS) [70, 71]. The 47-item self-report measure includes six subscales, with each item being presented on a 4-point Likert scale ranging from 0 (Never) to 3 (Always). Depression symptoms were assessed using the Low-Mood subscale. Scores were calculated by summing the items in the relevant subscale and converting them to T-scores based on the child’s gender and grade level. As grade level was not directly collected, it was retrospectively estimated using the state-recommended school enrolment age and the timing of study participation. T-scores of 65 or higher indicate borderline clinical levels, while scores of 70 or above reflect clinically significant symptomatology.

The German version of the Low-Mood subscale demonstrated good internal consistency (Cronbach’s *α* = 0.87) and strong convergent validity with the Depression Inventory for Children and Adolescents (DIKJ), *r* = .78 [71]. Anxiety symptoms were calculated using the same method [72]. However, the total anxiety score was derived from five subscales: social anxiety, generalised anxiety, separation anxiety, panic disorder, and obsessive-compulsive disorder. The R-CADS German version anxiety scales have shown excellent internal consistency (Cronbach’s α = 0.94) and strong correlations with both the Anxiety Questionnaire for Pupils (AFS; *r* = .81) [73] and the Spence Children’s Anxiety Scale (SCAS; *r* = .94) [74], indicating good convergent validity [71].

#### Childhood trauma

Childhood trauma was assessed using the German version of the Childhood Trauma Questionnaire (CTQ), a 28-item self-report measure of retrospective emotional, physical, and sexual abuse [75, 76]. The CTQ has demonstrated good psychometric properties in both clinical and community samples (77–79]. It was included due to established associations with trauma and altered salivary cortisol patterns [80].

### Preliminary analysis

#### Cortisol data imputation

An imputation strategy was applied to maximise participant retention in the cortisol analyses while minimising uncertainty. For missing T0 (*n* = 2), drooling test samples from the 30-minute pre-stressor acclimatisation period were substituted. Missing T1-T4 (*n* = 6) samples were estimated from surrounding timepoints. Two consecutive missing samples were generally not imputed, except in non-responders missing both T0 and T1, where T0 was substituted with the pre-stressor drooling test (*n* = 1).

#### Missing data for psychological instruments

No data were missing for IB or affective reactivity or recovery scores. One participant in the LR group did not complete the R-CADS anxiety and depression scales, two did not complete the CTQ, and three did not complete the SMRs. In the HR group, all questionnaires were completed. It is possible that missing data occurred only among LR participants because these questionnaires were administered at the end of the testing session, when discontinuing may have felt less consequential. By contrast, HR participants only proceeded to an app-based training after completing the questionnaires, which may have increased their motivation to remain until the end of the session. See RCT study protocol [53]. Participants with missing covariate data were excluded from analyses involving those variables. See Supplemental Materials Sect.  3 for information on the handling of outliers.

#### Computation of stress reactivity and recovery

Cortisol reactivity was defined as the highest value and affective reactivity as the lowest value observed between immediately after the stressor and 30 min later (+ 1, + 10, +20, or + 30 min), controlling for baseline levels. Higher cortisol scores indicated greater cortisol reactivity, whereas lower affect scores indicated greater mood reactivity. Recovery was defined as the value measured 45 min after the stressor for both cortisol and affect, controlling for baseline. For cortisol, higher values indicated worse recovery (i.e. cortisol levels had not returned to baseline). For affect, lower values indicate worse recovery (i.e. mood was still low and had not stabilised at the recovery time point). Initially, reactivity and recovery were defined as change scores (the pre-registered analysis plan can be accessed here: https://osf.io/tmf2v*).* However, because both baseline cortisol and affect were correlated with reactivity and recovery change scores (see Table S6 in the Supplemental Materials), we adapted our approach and instead used maximum cortisol / minimum affect and the 45-minute recovery markers directly, while including baseline scores as covariates. This strategy helped minimise baseline-related artefacts. For example, participants with higher baseline cortisol may appear to show better recovery simply because they have less scope for a further cortisol increase following the stressor (i.e. due to physiological limitations) and therefore less necessity for levels to decline thereafter, rather than because they are experiencing better recovery. Similarly, participants with lower initial affect ratings (worse mood) may appear to show greater improvement by the end of the testing period simply because they had more room to improve compared to participants who began with already high ratings (e.g. 8/9 mood rating) before the stressor (i.e. ceiling effect). Including baseline as a covariate rather than using change scores adjusts for these initial differences (see Supplemental Materials Sect.  2, Table S4-6, for original analyses with change scores). Moreover, computation approaches using peak cortisol for reactivity rather than area under the curve variations are consistent with recommendations [81, 82].

#### Validation of tanner sexual maturation rating

To evaluate the validity of SMR’s as a measure of pubertal status, we examined associations of self-reported pubertal stages with salivary hormone levels (log-transformed testosterone, estradiol, and progesterone). First, bivariate Pearson correlations were computed between SMR, age, and each hormone. Next, we conducted hierarchical linear regression analyses with age and then participants’ SMRs entered as predictors of each hormone outcome. This allowed us to test whether participants’ SMR accounted for unique variance in pubertal hormone levels above and beyond age.

Bivariate correlations showed that SMRs were significantly associated with testosterone (*r* = .50, *p* < .001) and progesterone (*r* = .33, *p* < .001), but not with estradiol (*r* = .17, *p* = .081). In addition, SMRs were significantly positively correlated with baseline cortisol (*r* = .31, *p* < .001). This pattern is consistent with prior evidence that basal cortisol levels increase with advancing pubertal stage. SMRs significantly predicted salivary testosterone and progesterone levels above and beyond chronological age, supporting their validity as indicators of pubertal development. No significant associations were found between SMRs or age and estradiol levels. Detailed regression results are provided in the Supplemental Materials, Sect.  3.

### Hypothesis testing

All statistical analyses were conducted in SPSS (Version 29). The analysis plan was uploaded to the OSF prior to data analysis (https://osf.io/tmf2v*).* Prior to hypothesis testing several preliminary analyses were conducted. Group differences in potential covariates (sex, SMR, RCADS depression and anxiety subscales and CTQ total score) were examined using independent-samples *t*-tests and chi-square tests (see Table 1). Pearson bivariate correlations were computed to examine associations between variables across the whole sample (see Table 2). To verify the effectiveness of the stress induction (TSST-C), one-sample *t*-tests were conducted across groups to examine whether self-reported or endocrinological stress reactivity change scores (i.e. peak cortisol increase and peak drop in affect up to 30-mins post stressor – baseline scores) significantly differed from zero.

#### Hypotheses 1 to 3

Group differences in IB, as well as in cortisol and affective reactivity and recovery, were tested using independent-samples *t*-tests and ANCOVAS for stress responses (where baseline scores were included as covariates) comparing HR and LR participants. Next, Bayesian *t*-tests and ANOVAS were calculated to warrant a rejection of null hypotheses. In addition, we used hierarchical multiple regression models to test whether group status explained unique variance beyond other relevant factors. For models predicting IB, symptom measures (RCADS depression and anxiety, CTQ) were entered in Step 1 and group status (HR vs. LR) in Step 2. For endocrinological outcomes, baseline cortisol, sex, and pubertal status were entered in Step 1, followed by symptom measures in Step 2 and group status in Step 3. For affective outcomes, only baseline affect was entered in Step 1, as sex and pubertal status were not expected to meaningfully influence these outcomes, followed by symptom measures in Step 2 and group status in Step 3.

### Hypothesis 4

Correlations (see Table 2) and partial correlations were computed to examine associations between IB and stress responses across the whole sample, and within each group separately, with partial correlations controlling for baseline cortisol or affect.

#### Exploratory analyses (Hypothesis 4 continued)

In line with theoretical accounts positing that cross-modal vulnerabilities will interact in shaping mental health outcomes [8, 9, 83], we conducted moderation analyses across the whole sample. Using the PROCESS macro (Model 1), we tested whether stress response (cortisol and affective reactivity and recovery) moderated the association between IB and symptoms of depression, controlling for baseline cortisol or affect. Given the significant correlations with baseline cortisol and depression as well as baseline affect and IB and depression (see Table 2), we also assessed models with baseline values as moderators.

## Results

### Descriptive statistics

See Table 1 for sample characteristics and Table 2 for correlations between study variables.

**Table 1:** Sample characteristics

Variable	Participants analysed per group (HR / LR)	HR	LR	Test statistic, *p*-value, and 95% CI
Sex: % female	*n* = 80 / 77	57.50	55.84	*χ²(*1) = 0.04, *p* = .83
Age: *M (SD)*	*n* = 80 / 77	12.03 (1.31)	12.45 (1.32)	*t*(155) = 2.03, *p* = .044*, 95% CI [0.01, 0.85]
DEP: *M (SD)*	*n* = 80 / 76	50.16 (9.47)	47.34 (8.61)	*t*(154) = − 1.95, *p* = .053, 95% CI [–5.68, 0.03]
ANX: *M (SD)*	*n* = 80 / 76	44.72 (9.88)	43.99 (8.36)	*t*(154) = − 0.50, *p* = .619, 95% CI [–3.63, 2.17]
CT: *M (SD)*	*n* = 80 / 75	31.84 (4.81)	31.52 (4.89)	*t*(153) = − 0.41, *p* = .684, 95% CI [–1.86, 1.22]
SMR: *M (SD)*	*n* = 80 / 74	2.69 (1.06)	2.91 (1.00)	*t*(152) = 1.31, *p* = .193, 95% CI [–0.11, 0.55]

*Note*.Sample sizes vary due to missing data. M (SD)= mean and standard deviations in brackets; DEP = depression symptoms (R-CADS Low Mood subscale); ANX = anxiety symptoms (mean of R-CADS anxiety subscales); CT = childhood trauma (Childhood Trauma Questionnaire); SMR = pubertal status (Tanner Sexual Maturation Ratings); HR = high-risk participants (children of parents with depression); LR = low risk participants (children of parents with no history of mental illness).

*p < .05.

**Table 2:** Correlations between study variables

	IB	B_Cort.	C_Reac.	C_Reco.	B_Aff.	A_Reac.	A_Reco.	DEP	ANX	CT	SMR	Sex
IB	—											
B_Cort.	0.02	—										
C_Reac.	− 0.09	0.46**	—									
C_Reco.	− 0.10	0.49**	0.92**	—								
B_Aff.	− 0.23*	− 0.002	0.09	0.09	—							
A_Reac.	− 0.24*	− 0.02	− 0.10	− 0.09	0.56**	—						
A_Reco.	− 0.16*	− 0.01	− 0.02	− 0.01	0.61**	0.43**	—					
DEP	0.55**	0.17*	0.03	0.06	− 0.33**	0.39**	− 0.22*	—				
ANX	0.50**	0.06	− 0.04	− 0.01	− 0.35**	0.38**	− 0.16	0.69**	—			
CT	0.47**	− 0.09	0.02	− 0.04	− 0.24**	− 0.23*	− 0.17*	0.44**	0.32**	—		
SMR	− 0.05	0.31**	0.17*	0.20*	0.01	− 0.04	− 0.06	0.09	0.02	− 0.16	—	
Sex	0.04	0.03	0.16*	0.16*	0.06	0.01	− 0.03	− 0.09	− 0.05	0.08	0.12	—

*Note*. Cortisol reactivity was denoted by the highest cortisol value observed following the stressor, and cortisol recovery by the cortisol value 45 min post-stressor. Affective reactivity was denoted by the lowest mood rating reported following the stressor, and affective recovery by the mood rating 45 min post-stressor. Elsewhere (i.e., in statistical models), reactivity and recovery refer to maximum cortisol/ minimum affect or 45-min values with baseline cortisol and affect included as covariates

IB = interpretation bias (Scrambled Sentences Task); B_Cort. = baseline cortisol; C_Reac. = cortisol reactivity; C_Reco. = cortisol recovery; B_Aff. = baseline affect (higher scores = better mood at baseline); A_Reac. = affective reactivity (reverse-coded, such that higher scores = greater reactivity); A_Reco. = affective recovery (higher scores = better mood at recovery); DEP = depression symptoms (R-CADS Low Mood subscale); ANX = anxiety symptoms (mean of R-CADS anxiety subscales); CT = childhood trauma (Childhood Trauma Questionnaire); SMR = pubertal status (Tanner Sexual Maturation Ratings); Sex = participant sex (0 = male, 1 = female)

*p < .05. **p < .001

### Normality testing

#### Interpretation bias

Shapiro-Wilk tests indicated that raw IB scores deviated from normality in both the LR, *W*[77] = 0.85, *p* < .001, and HR groups, *W*[80] = 0.80, *p* < .001. The arcsine-square-root transformation did not restore normality for either LR or HR groups respectively, *W*[77] = 0.95, *p* = .004, *W*[80] = 0.94, *p* < .001. Levene’s test supported the equal-variances assumption, *F*(1,155) = 0.01, *p* = .914. Because the transformation did not normalise the distributions and given comparable group sizes and the robustness of the independent-samples *t*-test, raw SST data were analysed.

#### Cortisol and affective reactivity and recovery

For standardised residuals of cortisol reactivity, *W*(156) = 0.97, *p* = .004, and recovery, *W*(151) = 0.97, *p* = .005, the Shapiro-Wilk test was significant. However, Levene’s test supported the equal-variances assumption for both reactivity, *F*(1,159) = 0.23, *p* = .635, and recovery, *F*(1,154) = 1.20, *p* = .276. Similarly, for standardised residuals of both affective reactivity, *W*(157) = 0.98, *p* = .021, and recovery, *W*(156) = 0.98, *p* = .021, the Shapiro-Wilk’s test was significant. However, Levene’s test supported the equal-variances assumption for both reactivity, *F*(1,155*)* = 1.91, *p* = .168, and recovery, *F*(1,154) = 1.54, *p* = .217. Deviations were not considered problematic for the planned analyses.

### Manipulation check

To verify the efficacy of the TSST-C, paired-samples t-tests compared baseline cortisol with peak cortisol and baseline affect with the lowest post-stressor affect rating across the full sample. Cortisol levels were significantly higher at peak than at baseline, *t*(155) = − 14.24, *p* < .001, *d* = 1.14, 95% CI [− 1.34, − 0.94], and mood ratings were significantly lower following the stressor, *t*(156) = 15.95, *p* < .001, *d* = 1.27, 95% CI [1.06, 1.48], confirming effective stress induction.

### Group comparisons (H1-H3)

An independent-samples *t*-test indicated no significant group difference in IB, and ANCOVAs revealed no significant group effects on stress responses controlling for baseline (see Table 3). A Bayesian independent samples *t*-test examining group differences in IB indicated moderate evidence supporting the absence of a group difference (BF₁₀ = 0.17). Bayesian ANCOVAs examining the effect of group on cortisol and subjective stress reactivity and recovery, while controlling for baseline levels, indicated moderate evidence supporting the absence of a group effect for cortisol reactivity (BF_incl = 0.23), cortisol recovery (BF_incl = 0.26), affective reactivity (BF_incl = 0.17) and affective recovery (BF_incl = 0.41).

**Table 3:** Group comparisons on outcome variables

Variable	Participants analysed per group (HR / LR)	HR M (SD)	LR M (SD)	Test statistic, *p*-value, and 95% CI
IB	*n* = 80 / 77	0.12 (0.13)	0.12 (0.12)	*t*(155) = 0.02, *p* = .982, 95% CI [–0.04, 0.04], *d* = 0.00
Cortisol reactivity	*n* = 79 / 77	1.51 (0.90)	1.40 (0.87)	*F*(1, 153) = 0.28, *p* = .599, partial *η*² = 0.00
Cortisol recovery	*n* = 78 / 73	0.87 (0.80)	0.75 (0.68)	*F*(1,148) = 0.91, *p* = .343, partial *η*² = 0.00
Affective reactivity	*n* = 80 / 77	5.77 (1.24)	5.96 (1.59)	*F*(1,154) = 0.01, *p* = .933, partial *η*² < 0.001
Affective recovery	*n* = 79 / 77	7.52 (1.24)	7.79 (1.01)	*F*(1,153) = 1.93, *p* = .167, partial *η*² = 0.01

*Note*. The reported means represent absolute observed values at each assessment time point and are not adjusted for baseline levels. For cortisol, higher reactivity and recovery scores reflect higher cortisol levels during the 30 minutes following the stressor and at the 45-minute recovery assessment, respectively. For affect, lower reactivity and recovery scores reflect lower mood at the corresponding time points. Statistical comparisons were conducted using analyses controlling for baseline levels

IB = interpretation bias (Scrambled Sentences Task); HR = high-risk participants (children of parents with depression); LR = low risk participants (children of parents with no history of mental illness)

### Regression models (Continuation of H1-H3)

#### Interpretation bias

At Step 1, depression symptoms, anxiety symptoms, and childhood trauma were entered, explaining 39.2% of the variance in IB, *F*(3, 151) = 32.49, *p* < .001. All three predictors emerged as significant unique contributors, with higher depression symptoms (*β* = 0.29, *p* = .002; See Figure S1 in Sect.  3 of the Supplemental Materials), anxiety symptoms (*β* = 0.21, *p* = .017), and childhood trauma (*β* = 0.28, *p* < .001) associated with stronger negative IB. At Step 2, familial risk status (HR vs. LR) was entered, but did not explain significant additional variance, *ΔR²* = 0.01, *ΔF*(1, 150) = 1.19, *p* = .277.

#### Cortisol and affective reactivity and recovery

Familial risk status did not predict cortisol or affective responses while accounting for baseline cortisol or affect and covariates (i.e., pubertal status, childhood trauma, depression and anxiety symptoms for cortisol models and childhood trauma, depression and anxiety symptoms for affective stress response models; see Supplemental Materials Sect.  3, Table S8). Across all stress response models, baseline cortisol and baseline affect showed significant main effects on reactivity and recovery scores (all *p*s < 0.001). Sex was a significant predictor of cortisol recovery in step 1 of the model and became insignificant when clinical variables were added in step 2.

### Associations between interpretation bias and stress responses (H4)

Interpretation bias was not significantly associated with baseline cortisol (see Table 2) and, in partial correlations controlling for baseline, was also unrelated to cortisol reactivity (*r* = –.10, *p* = .223) and recovery (*r* = –.11, *p* = .163) across the whole sample. Interpretation bias was, however, significantly associated with baseline affect across the whole sample (see Table 2), but in partial correlations controlling for baseline affect, it was not associated with affective reactivity (*r* = –.14, *p* = .081) or recovery (*r* = –.02, *p* = .772).

When looking at correlations between IB and stress responses within HR and LR groups individually we found no significant correlations between IB and baseline cortisol within either group (Hr_*r* = 0.06, *p* = .604, LR*_r* = –0.03, *p* = .821). In partial correlations controlling for baseline cortisol, there was a significant correlation between IB and cortisol reactivity (*r* = –.23, *p* = .042) as well as cortisol recovery (*r* = –.25, *p* = .031), within the HR group specifically. Within the LR group the effect for both reactivity, (*r* = .07, *p* = .588) and recovery (*r* = .07, *p* = .589) was non-significant.

In contrary, baseline affect was significantly correlated with IB in both the LR (*r* = –.24, *p* = .012) and HR (*r* = –.23, *p* = .037) groups. When baseline affect was controlled for, affective reactivity was no longer significantly correlated with IB in the LR (*r* = − .13, *p* = .251) or HR groups (*r* = − .14, *p* = .236). Affective recovery was also not associated with IB in LR (*r* = − .04, *p* = .710) or HR groups (*r* = − .01, *p* = .901), when controlling for baseline affect.

#### Exploratory moderation analyses

Moderation models were conducted to examine whether baseline, reactivity, and recovery indices of cortisol and affect moderated the association between IB and depressive symptoms. Interpretation bias significantly predicted depressive symptoms across all but one model. However, neither cortisol reactivity nor recovery, nor affective reactivity or recovery, significantly moderated the relationship between IB and depression. A significant interaction emerged only for baseline cortisol, indicating that higher baseline cortisol strengthened the association between IB and depressive symptoms, *ΔR²* = 0.03, *F*(1, 151) = 8.05, *p* = .005. Full model statistics are reported in the Supplementary Material (Sect.  3).

## Discussion

### Summary of findings

This is the first study to examine IB and endocrinological (cortisol) as well as self-reported (affect) stress responses to the TSST-C within the same sample of children at high and low familial risk for depression. Prior research has identified both negative IB [23, 84] and altered endocrinological stress [28] as key vulnerability factors, yet findings on cortisol responses (i.e. heightened vs. blunted reactivity and prolonged vs. steeper recovery) are inconsistent and little is known about how different vulnerabilities interact in HR youth. Contrary to hypotheses, HR and LR groups did not differ in IB, cortisol or affective reactivity or recovery post stress induction (H1-H3). Across both groups IB was most strongly associated with children’s current symptoms of depression. In partial support of our final hypothesis (H4), IB was associated with cortisol reactivity and recovery in HR participants only. Building on evidence that cross-modal vulnerabilities interact in shaping mental health outcomes [8, 9, 83], our exploratory analyses showed that baseline cortisol, but not cortisol or affective reactivity or recovery, moderated the association between IB and current depression symptoms.

#### Interpretation of findings

While group differences in IB, as measured by the SST, between HR and LR children reported in an earlier study could not be replicated [23], both that study and the present one found IB to be closely related to children’s own symptoms of depression. A further study using a mixed HR / LR sample, based largely on the same sample as the study mentioned in the previous sentence [23], found that IB predicted depression symptoms 30 months later, although not beyond baseline depression symptoms [13]. These findings suggest that IB may be closely tied to concurrent (subclinical) symptoms of depression, rather than representing a vulnerability that distinguishes HR from LR youth prior to the onset of symptoms. Thus, while IB may reflect a cognitive vulnerability relevant to depression, the present cross-sectional findings do not allow us to determine whether IB confers risk beyond concurrent subclinical symptoms, contributes to later depression indirectly through such symptoms, or is primarily an epiphenomenon of current depressive symptomatology.

In contrast to our findings on cortisol stress responses, a recent systematic literature review reported an overall tendency for HR and LR groups to differ in cortisol stress reactivity, yet also noted that findings from studies employing the TSST-C varied substantially [28]. This inconsistency is notable given that the TSST-C is widely regarded as a gold standard stress paradigm. One explanation is that reactions to social and performance challenges induced by the TSST-C differ from responses to other stress induction methods in HR populations, although the same review reported that social challenges were the most commonly used form of stress induction, with no uniform methodology across studies [28]. Furthermore, in comparison to a further meta-analysis of TSST studies in youth, which reported moderate effects for cortisol reactivity (*g* = 0.47) and affect (*g* = 0.57) [85], the present study found substantially larger effects for both cortisol (*d* = 1.14) and affective (*d* = 1.27) reactivity. These large effect sizes suggest that our null findings are unlikely to reflect insufficient stress induction and instead indicate genuine stress responses within the sample. Consistent with our results, a previous study [34] also reported no initial HR-LR differences in cortisol responses to the TSST-C, but found HPA axis blunting among HR adolescents when childhood trauma was taken into account. It is possible that risk status alone may not adequately explain alterations in stress responses. Instead, these findings highlight the importance of incorporating additional factors, such as trauma exposure, into theoretical models to clarify moderating mechanisms shaping stress responses.

Partially consistent with our final hypothesis, IB was associated with blunted stress reactivity and quicker return to baseline (recovery) in the HR sample (though not in the full sample). To the best of our knowledge, only one further study has simultaneously measured IB and cortisol responses to stress [48]. In line with our findings, the authors also found a blunted cortisol response to be associated with IB in their sample of adolescents with Autism Spectrum Disorder [48]. The observed pattern also aligns with evidence of HPA axis blunting in HR individuals exposed to elevated childhood trauma [45]. Although childhood trauma in our sample was linked to higher IB, it was not directly associated with cortisol reactivity or recovery. It is possible that IB may represent a more proximal mechanism connecting early adversity to habituation of stress responses. The finding is also consistent with theories proposing that the negative processing of a stressor shapes physiological stress responses, which then leads to symptoms of depression [9]. The finding that IB was associated with cortisol reactivity and recovery only in the HR group may reflect greater variability in this group, which could have increased the power to detect such effects.

Contrary to expectations, IB was unrelated to affective reactivity or recovery once baseline affect was controlled. However, IB was significantly associated with baseline affect across the full sample, such that a more negative IB corresponded to lower baseline affect. These findings are consistent with previous research (86–88]. Moreover, affective reactivity predicted depressive symptoms, which is also in line with previous prospective studies [89].

Finally, in line with model accounts which posit that cross-modal vulnerabilities interact in shaping mental health outcomes [90, 91], our exploratory moderation findings showed that heightened baseline cortisol strengthened the IB-depression relationship.

### Clinical and theoretical implications

Our findings indicate that parental depression status alone was not associated with the cognitive, affective, or endocrinological vulnerabilities assessed in this sample. At first glance, this appears inconsistent with familial transmission models [8], which posit that HR and LR groups can be differentiated in such vulnerabilities before the onset of symptoms. However, such models also emphasise that risk transmission is shaped by moderating factors, including the timing and course of maternal depression, as well as child characteristics such as temperament and social-cognitive skills. The present findings may therefore suggest that risk transmission does not operate through uniform elevations in isolated vulnerability domains, but rather through more complex and interacting processes that were not fully captured in the current study. Moreover, HR participants showed descriptively, though not significantly, higher depressive symptoms, and these symptoms were associated with vulnerabilities across cognitive (IB), biological (baseline cortisol), and affective (baseline affect and affective reactivity) domains. This pattern suggests that relevant vulnerabilities may have been present but too subtle to reliably distinguish groups in the current sample and may also have been more closely linked to current subclinical symptoms than to familial risk status alone.

Our findings provide preliminary support for theoretical accounts suggesting that cognitive, affective, and endocrinological vulnerabilities are interrelated [8, 9, 83], with IB linked to both cortisol reactivity and recovery and baseline affect and baseline cortisol moderating the IB-depression relationship. In particular, the observed association between IB and altered cortisol responses in HR youth is compatible with the perseverative cognition hypothesis, which posits that sustained negative cognitive processing can shape physiological stress activation. Although the present design does not allow conclusions about temporal sequencing, which should be addressed in future longitudinal research, these findings highlight the potential for cognitive processes to play a role in the regulation of stress physiology. Findings suggest that IB, independent of parental depression status, may represent a useful intervention target through its influence on endocrinological stress responses and close association with symptoms of depression and anxiety. However, rather than focusing on HR youth, preventive efforts might be most effective when directed toward individuals who have been found to exhibit a pronounced negative IB.

### Strengths, limitations and future directions

The study combined cognitive and physiological perspectives by assessing IB alongside both subjective and endocrinological stress indices in HR and LR youth within the same study. Use of a gold standard laboratory stressor (TSST-C) and parallel operationalisations of reactivity and recovery for cortisol and affective responses strengthened construct coverage and allows comparison between measures. Analytically, we differentiated reactivity and recovery from baseline scores allowing the investigation of diverse associations among stress parameters. The inclusion of baseline values as covariates for both endocrinological and subjective stress measures helped reduce measurement artefacts and enabled a more accurate estimation of stress reactivity and recovery. A final strength of this study is the assessment of pubertal status, with self-reported SMRs validated against salivary hormone.

The cross-sectional design precludes inferences about temporal precedence or causality; IB may precede symptoms, covary with them, or reflect their consequence at subclinical levels. Furthermore, we did not test higher-order interactions among variables or moderating factors, as this was beyond the scope of the study. The study may have benefited from assessing additional variables known to influence cortisol levels, such as menstrual cycle phase, where applicable. However, given the already substantial length of the testing sessions, the assessment battery was necessarily limited to minimise participant burden. Future research should build on these findings by experimentally manipulating IB in pre-screened samples with elevated IB to determine whether changes in IB patterns causally influence cortisol and affective stress reactivity. Finally, future studies should investigate moderators of the relationship between parental mental health status and vulnerability factors.

## Conclusion

This study is the first to examine IB alongside both cortisol and affective stress responses to the TSST-C in children at high and low familial risk for depression. Understanding how cognitive, affective and biological vulnerability factors interact in this group is important, given that existing preventive interventions for children of parents with depression show relatively modest effects [7]. We found no group differences in IB or stress responses, suggesting that the effects of parental depression in our sample may not be pronounced enough for children to exhibit the vulnerability characteristics investigated in this study. Instead, IB was closely associated with current symptoms of depression, to cortisol reactivity and recovery within the HR sample and baseline affect across the whole sample. Exploratory analyses further suggested that baseline cortisol moderated the IB-depression relationship. While this cross-sectional study offers an important foundation for integrating cognitive, affective, and endocrinological risk markers in youth, experimental research in samples where this bias is evident would be valuable for clarifying the causal influence of IB on stress responses.

## Supplementary Information

Below is the link to the electronic supplementary material.

Supplementary Material 1

## Data Availability

The datasets used and/or analysed during the current study are available from the corresponding author on reasonable request.

## Abbreviations

AB: Attentional Bias
AFS: Anxiety Questionnaire for Pupils
AUC: Area Under the Curve
CBM-I: Cognitive Bias Modification of Interpretations
CTQ: Childhood Trauma Questionnaire
DIPS: Diagnostic Interview for Mental Disorders
DSM-5: Diagnostic and Statistical Manual of Mental Disorders
EMA: Ecological Momentary Assessments
ES: Effect Size
HPA: Hypothalamic-pituitary-adrenal
HR: High-Risk (children of parents with depression)
IB: Interpretation Bias
K-DIPS: Diagnostic Interview for Mental Disorders in Children and Adolescents
LMU: Ludwig-Maximilians-University
LR: Low Risk (children of parents with no history of mental health issues)
MB: Memory Bias
RCT: Randomised Controlled Trial
R-CADS: Revised Children’s Anxiety and Depression Scale
SCAS: Spence Children’s Anxiety Scale
SMR: Tanner’s Sexual Maturation Rating
SST: Scrambled Sentences Task
TSST: Trier Social Stress Test
TSST-C: Trier Social Stress Test for Children
