# Supplementary material for: Investigating interpretation bias and stress responses as risk factors in children of parents with depression
Source: BMC Psychiatry. 2026 Jul 29;26:576. doi: 10.1186/s12888-026-08408-z (PMC13419086; doi:10.1186/s12888-026-08408-z)
Supplement: Supplementary file 1 — Supplementary Material 1 [file 12888_2026_8408_MOESM1_ESM.docx]

**Supplemental Materials**

**Section 1**

***English Translation of Scrambled Sentences***

**Table S1.1**

*Emotional Sentences Version A*

| I | always | things | never | accomplish | almost |
| --- | --- | --- | --- | --- | --- |
| total | I | winner | am | loser | a |
| do | people | dislike | me | like | usually |
| ideas | smart | I | have | dumb | often |
| things | bad | to | me | nice | happen |
| many | adorable | I | bad | have | traits |
| life | pretty | exciting | is | boring | my |
| fun | have | rarely | quite | often | I |
| I | pretty | hardly | concentrate | easily | can |
| I | others | laugh | than | cry | more |
| be | future | terrible | will | fantastic | my |
| my | good | life | bad | is | not |
| quite | are | ugly | my | pretty | looks |
| quite | sad | I | usually | happy | am |
| I | a | worthless | am | valuable | person |
| about | don't | care | friends | do | me |
| often | quite | do | succeed | not | I |
| a | I | good | impression | bad | leave |
| my | fail | will | goals | achieve | I |
| be | awesome | life | cruel | pretty | can |
| disappoint | often | family | rarely | I | my |
| give | up | never | will | eventually | I |
| my | good | is | mood | bad | usually |
| are | people | unfriendly | pretty | helpful | most |
| to | I | lots | have | nothing | offer |
| I | fit | usually | am | tired | quite |
| person | satisfied | I | dissatisfied | am | a |
| many | I | succeed | things | fail | in |
| I | always | am | never | sad | almost |
| others | I | inferior | am | superior | to |

*^Note. Scrambled emotional sentences used for Version A of the Scrambled Sentences Task (SST), translated from German to English.^*

**Table S1.2**

*Neutral Sentences Version A: Including Actual Trials and Three Demo Items*

| often | cinema | I | theatre | visit | the |
| --- | --- | --- | --- | --- | --- |
| my | is | pizza | favourite | fries | food |
| swimming | sometimes | going | I | occasionally | like |
| I | not | dogs | do | much | like |
| like | watching | funny | I | exciting | movies |
| I | holiday | swimming | on | hiking | like |
| television | comedies | on | thrillers | I | watch |
| to | I | late | bed | early | go |
| it | sunny | last | was | warm | summer |

*^Note. Scrambled neutral sentences used for Version A of the Scrambled Sentences Task (SST), translated from German to English.^*

**Table S2.1**

*Emotional Sentences Version B*

| I | master | life | ruin | my | will |
| --- | --- | --- | --- | --- | --- |
| very | am | lucky | I | unlucky | really |
| think | people | stupid | I | Nice | am |
| ideas | bad | often | have | good | I |
| experience | good | I | bad | things | often |
| am | inferior | others | likable | think | I |
| my | pretty | great | life | bad | is |
| fun | have | little | I | great | really |
| very | I | seldom | concentrate | often | well |
| than | others | giggle | I | cry | more |
| future | quite | bright | my | dark | looks |
| my | boring | life | interesting | pretty | is |
| I | person | interesting | a | boring | am |
| usually | unhappy | I | happy | am | pretty |
| am | I | worthless | person | useful | a |
| wellbeing | unimportant | find | my | important | others |
| be | fortunate | to | tend | misfortunate | I |
| a | leave | mediocre | I | brilliant | impression |
| important | skilfully | I | tasks | poorly | complete |
| my | fabulous | find | unbearable | I | life |
| by | disappointed | others | impressed | me | are |
| will | I | never | give | up | surely |
| feel | bad | quite | usually | good | I |
| people | most | mean | often | friendly | are |
| I | many | useful | have | useless | skills |
| typically | unwell | feel | I | well | very |
| a | joyful | person | miserable | I | am |
| am | typically | successful | very | unsuccessful | I |
| quite | cheerful | often | down | I | feel |
| by | feel | rejected | I | liked | others |

*^Note. Scrambled emotional sentences used for Version B of the Scrambled Sentences Task (SST), translated from German to English.^*

**Table S2.2**

*Neutral Sentences Version B: Including Actual Trials and Three Demo Items*

| is | summer | favourite | winter | season | my |
| --- | --- | --- | --- | --- | --- |
| like | I | sweets | a | cake | lot |
| like | funny | I | books | exciting | reading |
| really | modern | I | music | classical | like |
| like | I | early | getting | late | up |
| sweet | chocolate | my | favourite | cookies | is |
| in | occasionally | rains | sometimes | it | spring |
| eat | cereals | for | bread | breakfast | I |
| really | eating | vegetables | I | fruit | like |

*^Note. Scrambled neutral sentences used for Version B of the Scrambled Sentences Task (SST), translated from German to English.^*

**Section 2: Pre-Registered Analyses Using Originally Planned Change Scores and Investigating Responder Status**

While we had planned and pre-registered secondary analyses with cortisol responders only, defined as those showing a raw baseline-to-peak increase > 1.5 nmol/l (83), after adapting our analysis strategy, we refrained from conducting these secondary analyses as 67 participants (42.95% of the sample) would have been excluded, thus undermining the validity and utility of such analyses (see Supplemental Materials Section 2, Table S3, for details on responder status and group comparisons between responders and non-responders).

***Handling of Outliers***

One extreme outlier for affective recovery change scores (+4.49 *SD*) was identified and excluded. After computing cortisol reactivity and recovery change scores, one cortisol recovery score was identified as an outlier (–12.24 *SD*) and removed.

***Normality Assessment Affective Reactivity and Recovery***

Assumption testing for affective reactivity and recovery indicated violations of normality in both groups, as assessed by the Shapiro-Wilk test (reactivity: HR *W* = 0.94, *df* = 80, *p* < .001; LR *W* = 0.93, *df* = 77, *p* < .001; recovery: HR *W* = 0.89, *df* = 79, *p* < .001; LR *W* = 0.87, *df* = 77, *p* < .001). Levene’s tests were non-significant (reactivity: *F* = 1.09, *p* = .299; recovery: *F* = 0.23, *p* = 0.631.), indicating homogeneity of variances.

***Normality Assessment Physiological Stress Reactivity and Recovery***

### Log transformation was conducted to improve the symmetry of the distributions, and Shapiro-Wilk tests indicated that normality was met for most group × outcome combinations (all *p* > .05), with deviations only for cortisol reactivity in the LR group, *W*(73) = 0.95, *p* = .004. Levene’s test indicated that the assumption of homogeneity of variances was met for both outcomes (all *p* > .05).

***Responder Status***

There were no group differences between responders and non-responders (those who didn’t elicit a cortisol response greater than 1.5 nmol/l) in terms of group membership (HR vs. LR), sex, depression and anxiety symptoms or childhood trauma experience. See Table S1. There was a significant difference between responders and non-responders in affective reactivity, such that non-responders reported being less stressed than responders. However, cortisol non-responders still displayed subjective stress change scores which were significantly greater than zero, t(66) = 9.53, p < .001, 95% CI [1.03, 1.58], indicating that participants experienced a marked increase in subjective stress despite lacking a physiological cortisol response. This effect size was large, d = 1.17, 95% CI [0.85, 1.47].

**Table S3**

*Cortisol Responder Status*

| Variable | No. of participants analysed per group  (responders / non-responders) | Responders | Non-Responders | Test statistic, p-value, and 95% CI |
| --- | --- | --- | --- | --- |
| Group  % HR | *n* = 89 / 67 | 53.93% | 46.27% | *χ²(*1)= 0.90, *p* = .343 |
| Sex  % female | *n* = 89 / 67 | 61.80% | 50.75% | *χ²*(1)= 1.91, *p* = .167 |
| Age  *M* | *n* = 89 / 67 | 12.35 (1.40) | 12.06 (1.24) | *t*(154) = -1.34, *p* = .183, 95% CI [-0.72, 0.14] |
| DEP:  R-CADS  *M (SD)* | *n* = 88 / 67 | 48.81 (8.71) | 48.75 (9.80) | *t*(153) = -0.04, *p* = .483, 95% CI [–3.01, 2.88] |
| ANX:  R-CADS  *M (SD)* | *n* = 88 / 67 | 44.07 (8.45) | 44.77 (10.10) | *t*(153) = 0.46, *p* = .645, 95% CI [–2.26, 3.63] |
| CT:  CTQ *M (SD)* | *n* = 88 / 67 | 32.20 (4.85) | 31.08 (4.75) | *t*(152) = -1.44, *p* = .152, 95% CI [–2.68, 0.42] |

*^Note.^* *^M^* *^(SD)^* ^= mean and standard deviations in brackets; HR = high risk participants (children of parents with depression); DEP = depressive symptoms (R-CADS Low Mood Subscale); ANX = anxiety symptoms (mean of R-CADS Anxiety Subscales); R-CADS = Children’s Anxiety and Depression Scale; CT = childhood trauma (CTQ = Childhood Trauma Questionnaire).^

### ***Group Comparisons (H1-H3)***

### All independent-samples t-tests comparing HR and LR groups on IB as well as affective and cortisol stress reactivity and recovery (the latter examined in the full sample and in the cortisol responders-only subset) were non-significant (see Table S2).

### **Table S4**

*Group Comparisons on Outcome Variables Using Change Scores for Stress Indices*

| Variable | No. of participants analysed per group  (HR / LR) | HR | LR | Test statistic, *p*-value, and 95% CI |
| --- | --- | --- | --- | --- |
| IB | *n* = 80 / 77 | 0.12 (0.13) | 0.12 (0.12) | *t*(155) = 0.02, *p* = .982, 95% CI [-0.04, 0.04] |
| Affective Reactivity | *n* = 80 / 77 | 1.51 (1.31) | 1.56 (1.09) | *t*(155) = 0.27, *p* = .787, 95% CI [−0.33, 0.43]. |
| Affective Recovery | *n* = 79 / 77 | -0.19 (1.04) | -0.32 (0.92) | *t*(154) = -0.856, *p* = .394, 95% CI [−0.45, 0.18] |
| Cortisol Reactivity | *n* = 79 / 77 | 0.96 (0.84) | 0.90 (0.80) | t(154) = −0.39, p = .697, 95% CI [−0.31, 0.21] |
| Cortisol Recovery | *n* = 78 / 73 | 0.29 (0.68) | 0.24 (0.66) | t(149) = −0.50, p = .615, 95% CI [−0.27, 0.16] |
| Cortisol Reactivity (responders) | *n* = 48 / 41 | 1.46 (0.62) | 1.51 (0.53) | t(87) = 0.37, p = .712, 95% CI [−0.20, 0.29] |
| Cortisol Recovery (responders) | *n* = 46 / 39 | 0.67 (0.55) | 0.66 (0.53) | t(83) = −0.13, p = .898, 95% CI [−0.25, 0.22] |

*^Note^*^. HR = high risk participants (children of parents with depression); LR = low risk participants (children with parents who do not have a history of mental illness); IB = interpretation bias (Scrambled Sentences Task). Both affective reactivity and recovery change scores were reverse coded, such that higher scores indicate a greater drop in mood and poorer recovery, respectively.^

***Regression Models (Continuation of H1-H3)***

**Interpretation bias**: To examine whether familial risk status predicted negative IB beyond clinical covariates, hierarchical multiple regression analyses were conducted with IB scores as the dependent variable. At Step 1, depressive symptoms, anxiety symptoms, and childhood trauma were entered, explaining 39.2% of the variance in IB, *F*(3, 151) = 32.49, *p* < .001. All three predictors emerged as significant unique contributors, with higher depressive symptoms (*β* = .29, *p* = .002), anxiety symptoms (*β* = .21, *p* = .017), and childhood trauma (*β* = .28, *p* < .001) associated with stronger negative IB. At Step 2, familial risk status (HR vs. LR) was entered, but did not explain significant additional variance, *ΔR²* = .01, *ΔF*(1, 150) = 1.19, *p* = .277, and was not a significant predictor in the final model (*β* = −.08, *p* = .248).

**Affective and physiological stress reactivity and recovery**: None of the analyses testing whether familial risk status predicted affective or physiological stress responses above and beyond clinical and demographic variables were significant. Hierarchical multiple regressions were conducted separately for reactivity and recovery, and for the full sample versus cortisol responders only (for cortisol analyses; see Table S3).

**Table S5**

*Regression Models Predicting Outcome Variables from Covariates and Group Status Using Change Scores for Stress Indices*

|  | Overall Model | | | Change Statistic | | |
| --- | --- | --- | --- | --- | --- | --- |
|  | *df* | *R^2^* | *F* | *ΔR*² | *ΔF* | *β* |
| Interpretation Bias | | | | | | |
| Step 1  DEP  ANX  CT | 3 | .39 | 32.49 |  |  | .27**  .22*  .28** |
| Step 2  DEP  ANX  CT  Group  (HR vs. LR) | 4 | .40 | 24.70 | .01 | 1.19 | .29**  .21*  .28***  -.07 |
| Affective Reactivity | | | | | | |
| Step 1  DEP  ANX  CT | 3 | .03 | 1.57 |  |  | .02  .01  -.01 |
| Step 2  DEP  ANX  CT  Group  (HR vs. LR) | 4 | .03 | 1.28 | .00 | 0.43 | .02  .01  -.01  -.13 |
| Affective Recovery | | | | | | |
| Step 1  DEP  ANX  CT | 3 | .03 | 1.57 |  |  | .03  -.18  -.03 |
| Step 2  DEP  ANX  CT  Group  (HR vs. LR) | 4 | .04 | 1.34 | 0.004 | 0.65 | .01  -.17  -.03  .07 |
| Cortisol Reactivity (Whole Sample) | | | | | | |
| Step 1  Sex  SMR | 2 | .03 | 2.23 |  |  | .15  -.09 |
| Step 2  Sex  SMR  DEP  ANX  CT | 5 | .05 | 1.66 | 0.03 | 1.28 | .13  -.05  -.12  -.06  .15 |
| Step 3  Sex  SMR  DEP  ANX  CT  Group  (HR vs. LR) | 6 | .06 | 1.43 | 0.002 | 0.31 | .12  -.04  -.12  -.06  .15  .05 |
| Cortisol Recovery (Whole Sample) | | | | | | |
| Step 1  Sex  SMR | 2 | .02 | 1.50 |  |  | .13  -.08 |
| Step 2  Sex  SMR  DEP  ANX  CT | 5 | .03 | 0.87 | 0.01 | 0.46 | .11  -.06  -.12  -.003  .04 |
| Step 3  Sex  SMR  DEP  ANX  CT  Group  (HR vs. LR) | 6 | .03 | 0.78 | 0.002 | 0.36 | .11  -.06  -.12  .004  .04  .05 |
| Cortisol Reactivity (Responders) | | | | | | |
| Step 1  Sex  SMR | 2 | .05 | 1.99 |  |  | .05  -.20 |
| Step 2  Sex  SMR  DEP  ANX  CT | 5 | .10 | 1.74 | 0.05 | 1.55 | .03  -.17  .01  .01  .02 |
| Step 3  Sex  SMR  DEP  ANX  CT  Group  (HR vs. LR) | 6 | .10 | 1.46 | 0.001 | 0.12 | 0.03  -0.18  -0.17  -0.12  0.09  -0.04 |
| Cortisol Recovery (Responders) | | | | | | |
| *Step 1*  Sex  SMR | 2 | .02 | 0.61 |  |  | .02  -.12 |
| Step 2  Sex  SMR  DEP  ANX  CT | 5 | .06 | 1.00 | 0.04 | 1.18 | .000  -.15  -.02  -.14  -.09 |
| Step 3  Sex  SMR  DEP  ANX  CT  Group  (HR vs. LR) | 6 | .06 | 0.79 | 0.000 | 0.002 | -.001  -.15  -.02  -.14  -.09  .006 |

*^Note.^* ^DEP = depressive symptoms (R-CADS Low Mood Subscale); ANX = anxiety symptoms (mean of R-CADS Anxiety Subscales); R-CADS = Children’s Anxiety and Depression Scale; CT = childhood trauma (Childhood Trauma Questionnaire); SMR = pubertal status (Tanner Sexual Maturation Ratings); Group = high-risk and low-risk participants; *^*^p^* ^< .05. **^*^p^* ^< .01. ***^*^p^* ^< .001. Both affective reactivity and recovery change scores were reverse coded, such that higher scores indicate a greater drop in mood and poorer recovery, respectively.^

***Correlations Between Interpretation Bias and Stress Responses (H4):***

**There were no significant correlations between IB and stress responses across the whole sample (see Table S4). When looking at correlations between IB and stress responses within HR and LR groups individually we found the following:** Higher IB scores were significantly associated with lower cortisol response indices, including cortisol reactivity (r = –.24, p = .032) and recovery (r = –.28, p = .012) across the whole sample and reactivity (r = –.31, p = .034), and recovery (r = –.38, p = .010) within the responders only subset. Subjective measures were not significantly correlated with IB scores.

**Table S6**
*Correlations Between Study Variables Ssing Change Scores for Stress Indices*

|  | DEP | ANX | CT | IB | B_  Aff. | A_  Reac. | A_  Reco. | C_  Base._W | C_  Reac._W | C_  Reco_W | C_  Base._R | C_  Reac_R | C_  Reco_R | SMR | Sex |
| --- | --- | --- | --- | --- | --- | --- | --- | --- | --- | --- | --- | --- | --- | --- | --- |
| DEP | — |  |  |  |  |  |  |  |  |  |  |  |  |  |  |
| ANX | .69** | — |  |  |  |  |  |  |  |  |  |  |  |  |  |
| CT | .44** | .32** | — |  |  |  |  |  |  |  |  |  |  |  |  |
| IB | .55** | .50** | .47** | — |  |  |  |  |  |  |  |  |  |  |  |
| B_Aff. | -.33** | -.35** | -.24** | -.23** | — |  |  |  |  |  |  |  |  |  |  |
| A_Reac. | .17* | .14 | .06 | .08 | .23** | — |  |  |  |  |  |  |  |  |  |
| A_Reco. | -.11 | -.18* | -.08 | -.06 | .33** | -.16* | — |  |  |  |  |  |  |  |  |
| C_Base._W | .17* | .06 | -.09 | .02 | -.00 | -.00 | .01 | — |  |  |  |  |  |  |  |
| C_Reac._W | -.10 | -.10 | -.10 | -.11 | .10 | .20* | .09 | -.29** | — |  |  |  |  |  |  |
| C_Reco_W | -.10 | -.08 | .11 | -.12 | .10 | .15 | .13 | -.40** | .90** | — |  |  |  |  |  |
| C_Base._R | .18 | .12 | -.10 | .03 | -.08 | -.10 | -.14 | 1.00** | -.50 | -.48** | — |  |  |  |  |
| C_Reac_R | -.22* | -.19 | -.08 | -.11 | .21* | .20 | .18 | -.50** | 1.00** | .87** | -.50** | — |  |  |  |
| C_Reco_R | -.17 | -.19 | -.11 | -.19 | .28* | .17 | .28* | -.44** | .87** | 1.00** | -.44** | .87** | — |  |  |
| SMR | .11 | .04 | -.15 | .05 | -.02 | .06 | .06 | .29** | -.07 | -.07 | .26* | -.20 | -.12 | — |  |
| Sex | -.09 | -.05 | .08 | .04 | .06 | .04 | .08 | .03 | .15 | .12 | .10 | .06 | .02 | .10 | — |

*^Note.^* ^DEP = depressive symptoms; ANX = anxiety symptoms; CT = childhood trauma; IB = interpretation bias; B_Aff. = baseline affect (where higher scores = better mood); A_Reac. = affective reactivity (reverse coded, such that higher scores = greater reactivity); A_Reco. = affective recovery (reverse coded, such that higher scores = worse recovery); C_Base._W = baseline cortisol (whole sample); C_Reac._W = cortisol reactivity (whole sample); C_Reco._W = cortisol recovery (whole sample); C_Base._R = basline cortisol (responders only); C_Reac._R = cortisol reactivity (responders only); C_Reco._R = cortisol recovery (responders only). SMR = Tanner Sexual Maturation Rating. *^*^p^* ^< .05. **^*^p^* ^< .001.^

**Section 3**

***Handling of Outliers***

IB scores (proportion of negative sentences) were screened for extreme outliers (±3.29 *SD*). No outliers were identified and no participants scored below the pre-determined 50% accuracy rate of grammatically correct sentence formation on the SST; therefore, no cases were excluded from IB analyses.

Standardisation of raw cortisol values flagged five extreme cases (LR = 1, HR = 4), but these reflected plausible variability rather than measurement error and were therefore retained. Raw values were subsequently log-transformed.

For both cortisol and affective reactivity and recovery standardised residuals were subsequently checked for outliers. Outliers were removed if values exceeded ±3.29 *SD* and were likely to distort the data. Inspection of standardised residuals for cortisol and affective reactivity indicated no cases exceeded ±3.29 *SD*. One case exceeded the ±3.29 cutoff for standardised residuals of cortisol recovery (*z* = 3.74). However, Cook’s distance (0.08) and leverage (0.02) were well within acceptable limits (89). Therefore, it was retained in the analyses. Residuals for the affective recovery model also showed one case exceeding the ±3.29 cutoff (*z* = 3.59). This observation showed moderate Cook’s distance (0.36) but high leverage (0.07) and was excluded from analyses.

In the hormone data, one extreme outlier (*z* = –5.03) was identified and removed from the log transformed estradiol data.

***Regression Analyses for Validation of Pubertal Status***

Regression analyses predicting testosterone showed that while age significantly predicted testosterone in an initial step, *b* = 0.34, SE = 0.07, *β* = .44, *t*(109) = 5.17, *p* <.001, accounting for 20% of the variance, *R^2^* = 0.20, *F*(1,109 )= 26.73, *p* <.001, in a second step, when SMRs were added to the model, age was no longer a significant predictor, *b* =0.16, SE = 0.08, *β* = .21, *t*(108) = 1.94, *p* =.055. The final model explained variance to 27%, *ΔR^2^* = .07, *F*(1,108) = 10.99, *p* = .001, with SMRs significantly predicted testosterone, *b* = 0.35, SE = 0.10, β = .36, *t*(108) = 3.32, *p* =.001.

Similarly, in the model predicting progesterone, in the first step, age significantly predicted progesterone, *b* = 0.13, SE = 0.04, *β* = .28, *t*(109) = 3.03, *p* =.003, explaining 8% of the variance, *R^2^* = .08, *F*(1,109) = 9.21, *p* = .003. In the second step, SMRs were added to the model and significantly predicted progesterone, *b* = 0.16, SE = 0.07, *β* = .26, *t*(108) = 2.19, *p* = .031. The overall model was significant, *R^2^* = .12, F(2,108) = 7.15, *p* = .001. Age was no longer a significant predictor in the final model, *b* = 0.05, SE = 0.06, *β* = .11, *t*(108) = 0.92, *p* =.359.

In the model predicting estradiol, age was not a significant predictor, *b* = 0.08, SE = 0.05, *β* = .17, *t*(108) = 1.74, *p* = .085. In the second step, SMRs were added to the model but also did not significantly predict estradiol, *b* = 0.07, SE = 0.08, *β* = .10, *t*(107) = 0.84, *p* = .404. The overall model remained non-significant, R^2^ = .03, *F*(2,107) = 1.86, *p* = .161.

**Table S7**

*Overview of Screening and Outcome Measures*

| Purpose | Construct | Instrument / Response Indices |
| --- | --- | --- |
| Inclusion criteria | | |
|  | Parental diagnosis or no diagnosis | DIPS interview |
|  | Child no diagnosis | K-DIPS interview (parent + child report) |
| Outcome measures | | |
|  | Interpretation bias | SST (computer task) |
|  | Physiological stress response | Physiological stress reactivity:  Peak cortisol up to 30 minutes post-stressor with baseline cortisol as a covariate. Higher scores indicate greater reactivity relative to baseline. |
|  |  | Cortisol level 45 minutes after the stressor, with baseline cortisol included as a covariate. Higher scores indicate higher cortisol levels at recovery relative to baseline (i.e. worse recovery). |
|  | Affective stress response | Subjective stress reactivity:  The lowest mood rating on the SAM within 30 minutes after the stressor, where lower values indicate worse mood, with baseline mood included as a covariate. |
|  |  | Subjective stress recovery:  The SAM mood rating 45 minutes after the stressor, with baseline mood included as a covariate. |
| Confounding Variables | | |
|  | Pubertal status | SMR |
|  |  | Estradiol, progesterone and testosterone via drool assessment |
|  | Depression symptoms | R-CADS low mood subscale |
|  | Anxiety symptoms | R-CADS anxiety subscales averaged |
|  | Childhood trauma | CTQ total score |

*^Note.^*^CTQ = Childhood Trauma Questionnaire; DIPS = Diagnostic Interview for Mental Disorders; K-DIPS = Diagnostic Interview for Mental Disorders in Children and Adolescents; R-CADS = Children’s Anxiety and Depression Scale; SST = Scrambled Sentences Task, SAM = Self-Assessment Mannikin Scale; SMR = Tanner Sexual Maturation Rating.^

**Table S8**

*Regression Models Predicting Outcome Variables from Covariates and Group Status*

|  | Overall Model | | | Change Statistic | | |
| --- | --- | --- | --- | --- | --- | --- |
|  | *df* | *R2* | *F* | *ΔR*² | *ΔF* | *β* |
| Interpretation bias | | | | | | |
| Step 1  DEP  ANX  CT | 3 | .39 | 32.49 |  |  | .27**  .22*  .28** |
| Step 2  DEP  ANX  CT  Group | 4 | .40 | 24.70 | 0.01 | 1.19 | .29**  .21*  .28***  -.07 |
| Cortisol reactivity | | | | | | |
| Step 1  Baseline cortisol  Sex  SMR | 3 | .23 | 14.71 |  |  | .44**  .14  .02 |
| Step 2  Baseline cortisol  Sex  SMR  DEP  ANX  CT | 6 | .24 | 7.63 | 0.01 | 0.65 | .46**  .13  .04  -.03  -.07  .10 |
| Step 3  Baseline cortisol  Sex  SMR  DEP  ANX  CT  Group | 7 | .24 | 6.49 | 0.003 | 0.50 | .46**  .12  .05  -.04  -.07  .10  .05 |
| Cortisol recovery | | | | | | |
| Step 1  Baseline cortisol  Sex  SMR | 3 | .25 | 16.25 |  |  | .46**  .15*  .04 |
| Step 2  Baseline cortisol  Sex  SMR  DEP  ANX  CT | 6 | .25 | 7.99 | 0.001 | 0.05 | .46**  .14  .04  -.001  -.03  .000 |
| Step 3  Baseline cortisol  Sex  SMR  DEP  ANX  CT  Group | 7 | .26 | 6.98 | 0.005 | 0.92 | .46**  .14  .05  -.02  -.02  .007  .07 |
| Affective reactivity | | | | | | |
| Step 1  Baseline affect | 1 | .32 | 70.85 |  |  | .56** |
| Step 2  Baseline affect  DEP  ANX  CT | 4 | .37 | 22.01 | 0.05 | 4.24 | .47**  -.16  -.12  -.01 |
| Step 3  Baseline affect  DEP  ANX  CT  Group | 5 | .37 | 17.52 | 0.00 | 0.08 | .47**  -.16  -.10  -.01  .02 |
| Affective recovery | | | | | | |
| Step 1  Baseline affect | 1 | .38 | 93.32 |  |  | .62** |
| Step 2  Baseline affect  DEP  ANX  CTQ | 4 | .39 | 23.40 | 0.01 | 0.44 | .63**  -.07  .10  -.01 |
| Step 3  Baseline affect  DEP  ANX  CT  Group | 5 | .39 | 19.01 | 0.01 | 1.23 | .62**  -.05  .09  -.01  -.07 |

***^Note.^*** ^DEP = depression symptoms (R-CADS Low Mood Subscale); ANX = anxiety symptoms (mean of R-CADS Anxiety Subscales); CT = childhood trauma (Childhood Trauma Questionnaire); SMR = pubertal status (Tanner Sexual Maturation Ratings); Group = high-risk and low-risk participants; *^*^p^* ^< .05. **^*^p^* ^< .001.^

***Exploratory Moderation Analyses***

The overall moderation model examining whether cortisol reactivity moderated the relationship between IB and depression symptoms, controlling for baseline cortisol was significant, *F*(4, 150) = 18.25, *p* < .001, *R²* = .33. However, the IB × cortisol reactivity interaction was not significant, *ΔR²* < .001, *F*(1, 150) = 0.08, *p* = .778. The overall model with cortisol recovery as a moderator was also significant, *F*(4, 145) = 18.65, *p* < .001, *R²* = .34. However, the IB × cortisol recovery interaction was again not significant, *ΔR²* = .002, *F*(1, 145) = 0.48, *p* = .491. The overall model testing baseline cortisol as a moderator was significant, *F*(3, 151) = 28.44, *p* < .001, *R²* = .36, with the IB × baseline cortisol interaction also being significant, *ΔR²* = .03, *F*(1, 151) = 8.05, *p* = .005, indicating a significant moderation effect where higher baseline cortisol strengthened the relationship between IB and depression.

The overall model with affective reactivity as a moderator was significant, *F*(4, 151) = 22.84, *p* < .001, *R*² = .38. The IB × affective reactivity interaction was not significant, *ΔR*² < .001, *F*(1, 151) = 0.08, *p* = .780, indicating no moderation effect. The overall model with affective recovery was significant, *F*(4, 150) = 20.84, *p* < .001, *R²* = .36. The IB × affective recovery interaction was not significant, *ΔR²* = .001, *F*(1, 150) = 0.24, *p* = .627, indicating no moderation effect. The overall model with baseline affect as a moderator was again significant, *F*(3, 152) = 27.16, *p* < .001, *R²* = .35. The IB × baseline affect interaction was also not significant, *ΔR²* = .006, *F*(1, 152) = 1.32, *p* = .252, indicating no moderation effect. See Table S9 for regression coefficients of individual variables for moderation models.

# **Table S9** *Regression Coefficients for Moderation Models Predicting Depressive Symptoms*

| Model | Predictor | *b* | SE | *t* | *p* |
| --- | --- | --- | --- | --- | --- |
| Cortisol reactivity | Interpretation bias | 41.93 | 9.99 | 4.20 | <.001 |
|  | Cortisol reactivity | 0.25 | 1.09 | 0.23 | .818 |
|  | Baseline cortisol | 2.22 | 1.08 | 2.05 | .042 |
| Cortisol recovery | Interpretation bias | 44.23 | 7.32 | 6.04 | <.001 |
|  | Cortisol recovery | 0.98 | 1.27 | -0.69 | .490 |
|  | Baseline cortisol | 2.38 | 1.13 | 2.10 | .037 |
| Baseline cortisol | Interpretation bias | 27.75 | 6.24 | 4.44 | <.001 |
|  | Baseline cortisol | -0.57 | 1.35 | -0.42 | .672 |
| Affective reactivity | Interpretation bias | 38.45 | 16.42 | 2.34 | .020 |
|  | Affective reactivity | -1.56 | 0.67 | -2.34 | .020 |
|  | Baseline affect | -0.77 | 0.67 | -1.15 | .250 |
| Affective recovery | Interpretation bias | 48.73 | 27.61 | 1.76 | .080 |
|  | Affective recovery | 0.31 | 0.82 | 0.37 | .708 |
|  | Baseline affect | -2.14 | 0.74 | -2.89 | .004 |
| Baseline affect | Interpretation bias | 76.00 | 35.11 | 2.16 | .032 |
|  | Baseline affect | -1.14 | 0.80 | -1.42 | .159 |

*^Note.^* ^Whole model statistics and interaction effects (IB × moderator) are reported in the text.^

**Fig. S1:** Correlation between interpretation bias and symptoms of depression across the whole sample**.**

*Note.* B **=** Interpretation Bias (Scrambled Sentences Task)
